# Effect of Coca-Cola on the Dissolution of Persimmon-Related Phytobezoar in a Tertiary Care Hospital

**DOI:** 10.7759/cureus.54420

**Published:** 2024-02-18

**Authors:** Maryam Riaz, Ismail Akbar, Rao E Hassan, Waheed Ahmad, Hassamullah Khan, Arif Ullah Khan, Muhammad Hassan Khan, Syeda Sunaina Shah, Asna Tahir, Safina Tanveer

**Affiliations:** 1 Surgery, Ayub Teaching Hospital, Abbottabad, PAK; 2 Orthopedics and Trauma, Khyber Teaching Hospital-Medical Training Institute (MTI), Peshawar, PAK; 3 General Surgery, Hayatabad Medical Complex-Medical Training Institute (MTI), Peshawar, PAK; 4 Surgery, Lady Reading Hospital-Medical Training Institute (MTI), Peshawar, PAK; 5 Ophthalmology, Khyber Teaching Hospital-Medical Training Institute (MTI), Peshawar, PAK; 6 Surgery, Khyber Teaching Hospital-Medical Training Institute (MTI), Peshawar, PAK

**Keywords:** gastric bezoars, bezoars, diospyrobezoars, gastric lavage, efficacy, coca–cola, persimmon, phytobezoar

## Abstract

Introduction

Bezoars, masses of indigestible foreign bodies formed in the gastrointestinal tract, pose challenges in their management. Phytobezoars are particularly problematic due to their difficult diagnosis and resilience towards treatment. Recently, Coca-Cola has emerged as a potential solution due to its acidic composition and mucolytic properties. However, existing evidence is limited, highlighting the need for comprehensive studies. This research explores the efficacy of Coca-Cola in dissolving persimmon-related phytobezoars, aiming to contribute valuable insights to non-invasive treatment options.

Material and methods

Conducted as a descriptive case series, this study employed gastric cola lavage using non-probability purposive sampling. Patients aged 18-70 with persimmon-related phytobezoars were included. Two nasogastric tubes were inserted for cola lavage over 12 hours, utilizing three liters of cola until the disappearance of symptoms. When the bezoar disappeared, it was considered as complete success to the treatment.

Results

Out of 31 patients, 45.2% were male and 54.8% were female, with a mean age of 56.77 ± 9.01 years. Efficacy was noted in 54.8% of cases. Age less than 50 and no history of diabetes mellitus were associated with higher chances of treatment success (p-value ≤0.05).

Conclusion

Ingestion of Coca-Cola was highly effective, safe, and reliable for the dissolution of persimmon-related phytobezoars, as the frequency of efficacy was high in our study. Coca-Cola ingestion is a non-invasive and cost-effective mode of phytobezoar dissolution that should be taken as a first-line initial treatment option to attain desired outcomes.

## Introduction

Bezoars are foreign bodies formed in the gastrointestinal tract, often hard masses in the stomach comprised of animal or vegetable materials [1]. Their estimated occurrence is around 0.4% [2]. Conditions such as gastric dysmotility from diabetic gastroparesis or prior gastric surgery can elevate the risk of bezoar formation. Bezoars are categorized into five types based on their composition: phytobezoars, trichobezoars, medication bezoars, lactobezoars, and food bolus bezoars. Phytobezoars are mainly made up of indigestible cellulose, tannin, and lignin from vegetables and fruits. In certain Asian countries, persimmon and pineapple ingestion leads to diospyrobezoars, which are challenging to treat due to their hard consistency [3].

Managing phytobezoars typically involves conservative approaches such as administering proteolytic enzymes, cellulase, and carbonated beverages orally or through gastric lavage, sodium bicarbonate (NaHCO_3_) powder, aspiration, endoscopic fragmentation, and, in refractory cases, surgery [4,5]. The efficacy of these treatments varies significantly, especially in diospyrobezoars, which are implicated in some countries as a major etiological agent for phytobezoar development. Treating phytobezoars with hard consistency presents challenges, and procedures like endoscopic therapy with fragmentation or enzymatic dissolution may not always succeed [6].

Recently, cola has emerged as an effective treatment for dissolving gastric phytobezoars. However, reports on its efficacy are mostly limited to individual cases or small case series. The proposed mechanism involves the acidic nature of Coca-Cola, the mucolytic effect of sodium bicarbonate (NaHCO_3_) in the beverage, and carbon dioxide (CO_2_) bubbles penetrating the bezoar's surface, resulting in breaking down the concrete's fibers. Importantly, this treatment doesn't require specialized equipment or advanced endoscopic techniques, making it a viable option, especially for elderly patients [7,8].

To better comprehend the effectiveness of cola in phytobezoar dissolution, larger-scale studies with more extensive sample sizes are necessary. This research aims to determine the efficacy of Coca-Cola in phytobezoar dissolution, filling a gap in the research landscape and addressing the current magnitude of the local problem.

## Materials and methods

This descriptive case series was conducted at the Department of General Surgery, Ayub Teaching Hospital, Abbottabad, spanning from January 15 to July 15, 2023. The research focused on 31 patients diagnosed with persimmon-related phytobezoars. Ethical clearance for the study was obtained from the Medical Teaching Institute Abbottabad Institutional Ethical Review Committee (Approval number: RC-EA-2023/012), and participants provided written informed consent. The sampling strategy employed non-probability purposive sampling, including the patients diagnosed with persimmon-related gastric phytobezoar, aged 18-70 years, regardless of gender, who had ingested persimmons within the last three months in the study. Exclusion criteria were applied to individuals with a history of prior treatment for phytobezoar (endoscopic intervention), hepatic and stomach malignancies, and alcohol abuse.

**Figure 1:**
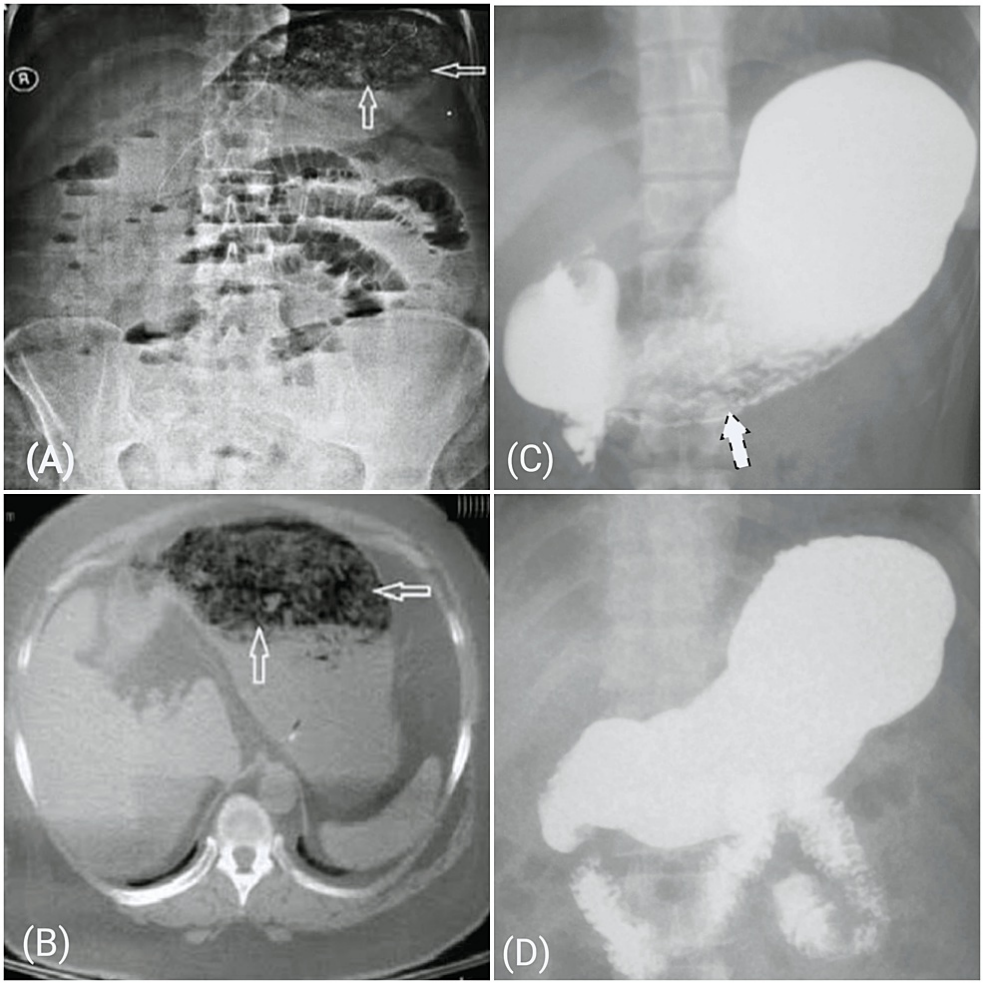
Erect abdomen x-ray (A) shows a shadow in the stomach (black arrows) and distended distal bowel; CT scan (B) shows a characteristic mottled appearance in the stomach; barium meal shows filling defect (C); and barium meal shows no filling defect and free passage of contrast in distal bowel (D) in a patient diagnosed with gastric outlet obstruction secondary to persimmon-related gastric phytobezoar before (A)-(C) and after (D) intervention. CT: computed tomography.

**Figure 2:**
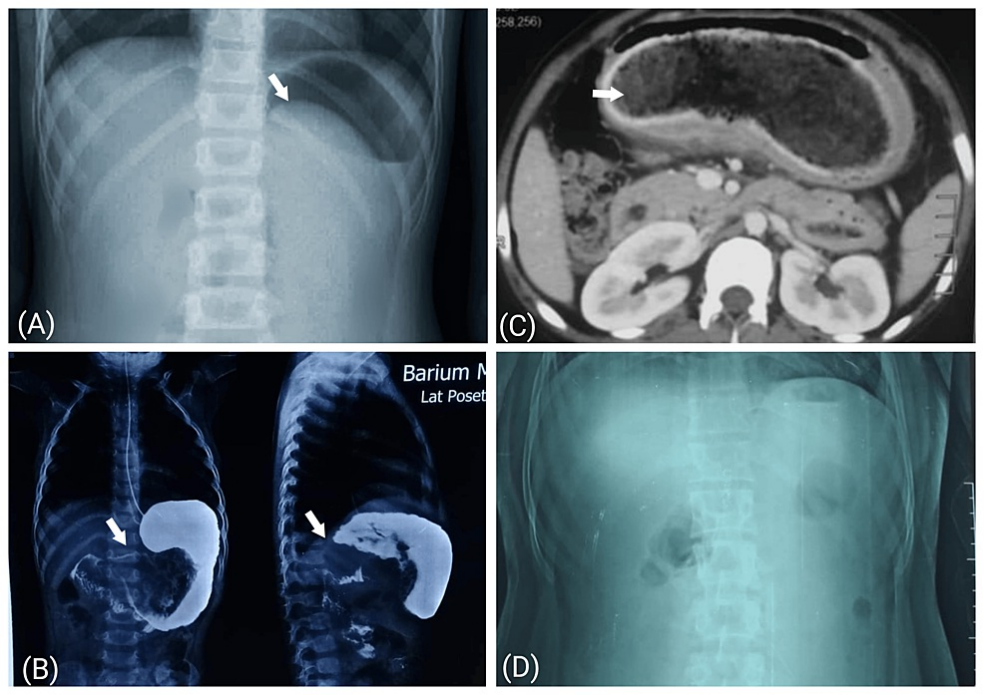
Erect abdomen X-ray showing ambiguous shadow (A) in the epigastrium and left hypochondrium (white arrow), barium meal (B); showing a filling defect in the gastric region, CT-scan (C); showing typical mottled appearance, and X-ray erect abdomen showing no significant findings (D) in a patient diagnosed with persimmon-related phytobezoar before (A)-(C) and after (D) intervention. CT: computed tomography.

Upon confirming the diagnosis of the persimmon-related gastric phytobezoar through history, examination, and diagnostic imaging (x-ray, ultrasonography, barium studies, and computed tomography), the intervention involved the use of two nasogastric tubes (16 Fr) for gastric cola lavage. One tube administered cola continuously, while the other facilitated natural drainage. The lavage process spanned 12 hours, utilizing three liters of cola. Diabetic patients received a 'Coca-Cola diet' with aspartame as a sweetener and no natural carbohydrates. Hourly blood glucose monitoring by a glucometer was done in all patients. Treatment progress was monitored for one day after the three-liter cola lavage. The improvement in signs and symptoms related to phytobezoar, along with supporting imaging findings like the alleviation of gastric distention and the disappearance of a mass or mobile intraluminal gastric filling defect, along with the disappearance of air-fluid levels and unidirectional gut peristalsis in cases of obstruction, signified the effective dissolution of the bezoar. Data entry was meticulously managed through a digital proforma under the researcher's supervision (Figures 1, 2). Patients who did not respond to cola lavage underwent upper gastrointestinal endoscopy for phytobezoar removal. Those who didn't improve to endoscopy proceeded to surgical intervention.

IBM SPSS Statistics for Windows, version 24.0 (released 2021; IBM Corp., Armonk, New York, United States) facilitated the entry and analysis of all collected data. Means and standard deviations were calculated for quantitative variables such as age and body mass index (BMI), while categorical variables such as gender, age groups, obesity, diabetes, hypertension, residential status, literacy, and efficacy were presented as frequencies and percentages. The chi-square test served to evaluate the impact of these variables on treatment responsiveness, with a p-value ≤0.05 considered statistically significant (95% confidence interval).

## Results

Thirty-one patients with persimmon-related phytobezoar participated in the study. The gender distribution comprised 45.2% (n = 14) males and 54.8% (n = 17) females. The mean age of these patients was 56.77 ± 9.01 years (range; 40-70 years), with 71% (n = 22) aged over 50 years. Illiteracy was observed in 64.5% (n = 20), and 51.6% (n = 16) hailed from rural areas. Diabetic patients constituted 25.8% (n = 8), while 45.2% (n = 14) had hypertension. The mean BMI was 24.35 ± 2.12 kg/m², with 16.1% (n = 5) classified as obese (BMI >30). Efficacy was noted in 54.8% (n = 17) of study cases. Patients who showed no improvement following cola lavage underwent endoscopic intervention, with one of them necessitating surgical intervention thereafter.

Upon applying the Chi-square test, it was established that individuals under 50 years of age and those without a history of diabetes mellitus (DM) showed a greater likelihood of treatment success, with a p-value ≤0.05. In contrast, all other variables displayed independence from treatment efficacy with a p-value >0.05 (Table 1).

**Table 1:** Cross-tabulation of the response of persimmon-related phytobezoar to the treatment with Coca-Cola with study variables showing statistically significant differences in response by age groups (above and below 50 years) and presence or absence of diabetes mellitus. The chi-square test was used with a p-value ≤0.05 significant.

Characteristics	Efficacy		P-value
	Yes (n = 17)	No (n = 14)	
Gender			
Male (n = 14)	08 (47.1%)	06 (42.9%)	0.815
Female (n = 17)	09 (52.9%)	08 (57.1%)	
Age groups			
Up to 50 years (n = 09)	08 (47.1%)	01 (7.1 %)	0.015
>50 Years (n = 22)	09 (52.9%)	13 (92.9%)	
Residential status			
Rural (n = 16)	09 (52.9%)	07 (50%)	0.870
Urban (n = 15)	08 (47.1%)	07 (50%)	
Diabetes mellitus			
Yes (n = 08)	02 (11.8%)	06 (42.9%)	0.049
No (n = 23)	15 (88.2%)	08 (57.1%)	
Hypertension			
Yes (n = 14)	06 (35.3%)	08 (57.1%)	0.224
No (n = 17)	11 (64.7%)	06 (42.9%)	
Obesity			
Yes (n = 05)	02 (11.8%)	03 (21.4%)	0.467
No (n = 26)	15 (88.2%)	11 (78.6%)	
Literacy			
Yes (n = 20)	10 (58.8%)	10 (71.4%)	0.465
No (n = 11)	07 (41.2%)	04 (28.6%)	

## Discussion

Coca-Cola is now commonly used as an initial treatment for uncomplicated gastric bezoars, particularly phytobezoars, through chemical dissolution [9]. The precise mechanism by which carbonated soft drinks dissolve bezoars remains unclear. It is hypothesized that the mucolytic properties of sodium bicarbonate and the acidifying effects of carbonic acid and phosphoric acid contribute to breaking down the bezoars. Additionally, the carbon dioxide bubbles in Coca-Cola permeate the bezoar fibers, enhancing the available surface area for the reaction to occur [1,7,8,10].

Thirty-one patients with persimmon-related phytobezoar were included in this study. The cohort comprised 45.2% (n = 14) male patients, while 54.8% (n = 17) were female patients. Notably, Toka et al. from Turkey documented 56.8% of male patients with a history of persimmon-related phytobezoar [11]. Similarly, Krausz et al., in a study across 12 major hospitals in Israel, reported 69% of male patients with a history of persimmon phytobezoars [12]. On the contrary, Ladas et al. observed a higher prevalence of persimmon-related phytobezoars in female patients, accounting for 55% of cases [8]. These varying findings suggest no clear gender predominance in cases of persimmon-related phytobezoars.

The mean age of patients with persimmon-related phytobezoar was 56.77 ± 9.01 years (range: 40-70 years), with 71% (n = 22) aged over 50 years. Among them, 64.5% were illiterate, and 51.6% (n = 16) resided in rural areas. Comparative studies by Toka et al. recorded a mean age of 57.6 ± 12.5 years for patients with a history of persimmon-related phytobezoar [11], while Krausz et al. reported a mean age of 46.8 years for patients with a history of persimmon phytobezoars [12]. Additionally, Ladas et al. observed a mean age of 60 years (range: 25-87 years) for persimmon-related phytobezoars, aligning with our findings [8]. This underscores the association between increasing age and the likelihood of developing persimmon-related phytobezoars. In our study, patients younger than 50 showed a higher propensity for treatment success compared to their older counterparts. To fully understand this phenomenon, more extensive studies are required.

Eight individuals (25.8%) had diabetes, and 45.2% (n = 14) were hypertensive. Toka et al. reported a history of diabetes and hypertension in 13.5% and 35.1% of patients with a history of persimmon-related phytobezoar, respectively [11]. In this study, patients without a history of diabetes were more likely to respond successfully to treatment than diabetic patients. This could be attributed to gastric dysmotility resulting from diabetic gastroparesis, a known predisposing factor for phytobezoar formation [3,13]. However, further, large-scale studies are required to confirm this relationship.

Coca-Cola is a non-invasive method for dissolving phytobezoars, offering a viable treatment option. It can be considered as an initial therapeutic approach in cases without clear indications of complete obstruction, bleeding, or perforation. This is particularly relevant in specialized healthcare facilities with prompt access to gastroenterology and surgical support. Physicians should be cautious of the potential risk of partially dissolved bezoars causing small intestinal obstructions during the treatment process [4]. Efficacy was noted in 54.8% (n = 17) of our study cases. Ladas et al. documented 50% efficacy with Coca-Cola ingestion alone and 91% with a combination of Coca-Cola and endoscopy [8]. However, endoscopy results were not included in our study, focusing solely on Coca-Cola dissolution of phytobezoars. Nelson et al. also demonstrated the superior efficacy of Coca-Cola in phytobezoar dissolution [14].

The potential adverse effects of Coca-Cola gastric lavage for phytobezoars remain understudied in the literature. Two documented side effects include gastric outlet obstruction and secondary ileus [10,15,16]. It is hypothesized that the CO_2_ bubbles released from Coca-Cola might elevate stomach pressure, facilitating the passage of the shrunken bezoar through the pylorus and potentially causing gastric outlet obstruction at the pylorus or elsewhere in the small intestine, leading to small bowel obstruction-mediated ileus [10,16]. Notably, no such incidents were observed during this study, possibly because of the prolonged lavage period of over 12 hours, allowing ample time for the dissolution of the bezoar. One of the recognized side effects of Coca-Cola (both regular and diet) is a decrease in serum high-density lipoprotein cholesterol and an increase in serum total cholesterol and low-density lipoprotein cholesterol [17]. Considering the one-time use of Coca-Cola in this study, these risks were deemed negligible.

The study's limitations include a small sample size of 31 patients from a specific hospital and limited geographic representation, reducing generalizability. Its descriptive case series design lacks a control group or randomization, hindering the establishment of a direct causal link between Coca-Cola ingestion and phytobezoar dissolution. While the study identifies associations between age, diabetes, and treatment success, it doesn't thoroughly investigate underlying mechanisms or potential confounding factors. Future research with larger samples, robust designs, and consideration of confounders is essential for validating and extending these findings.

## Conclusions

Our study underscores the potential effectiveness of Coca-Cola in addressing phytobezoars related to persimmon ingestion, especially among younger individuals without diabetes. While our results indicate that Coca-Cola consumption is highly efficient, safe, and reliable in dissolving these bezoars, caution is warranted in selecting suitable candidates for this intervention. Nonetheless, the correlation observed between the effectiveness of Coca-Cola and age groups and diabetic status highlights its promising utility as a non-invasive and cost-effective therapeutic option. However, further extensive research is imperative to validate and build upon these preliminary findings, ultimately advocating for the integration of Coca-Cola as a practical and accessible treatment in clinical settings.
